# Supplementary material for: Homogeneous versus Inhomogeneous Polarization Switching in PZT Thin Films: Impact of the Structural Quality and Correlation to the Negative Capacitance Effect
Source: Nanomaterials (Basel). 2021 Aug 20;11(8):2124. doi: 10.3390/nano11082124 (PMC8402043; doi:10.3390/nano11082124)
Supplement: Supplementary file 1 [file nanomaterials-11-02124-s001.zip › nanomaterials-1339811-supplementary.pdf]

nanomaterials

Supplementary Materials

Homogeneous versus Inhomogeneous Polarization Switching in PZT Thin Films: Impact of the Structural Quality and Correlation to the Negative Capacitance Effect

Lucian Pintilie *, Georgia Andra Boni, Cristina Florentina Chirila, Viorica Stancu, Lucian Trupina, Cosmin Marian Istrate, Cristian Radu and Ioana Pintilie

National Institute of Materials Physics, Atomistilor 405A, 077125 Magurele, Romania; andra.boni@infim.ro (G.A.B.); dragoi@infim.ro (C.F.C.); stancu@infim.ro (V.S.); Lucian.Trupina@infim.ro (L.T.); cosmin.istrate@infim.ro (C.M.I.); cristian.radu@infim.ro (C.R.); ioana@infim.ro (I.P.)
* Correspondence: pintilie@infim.ro

1. TEM studies

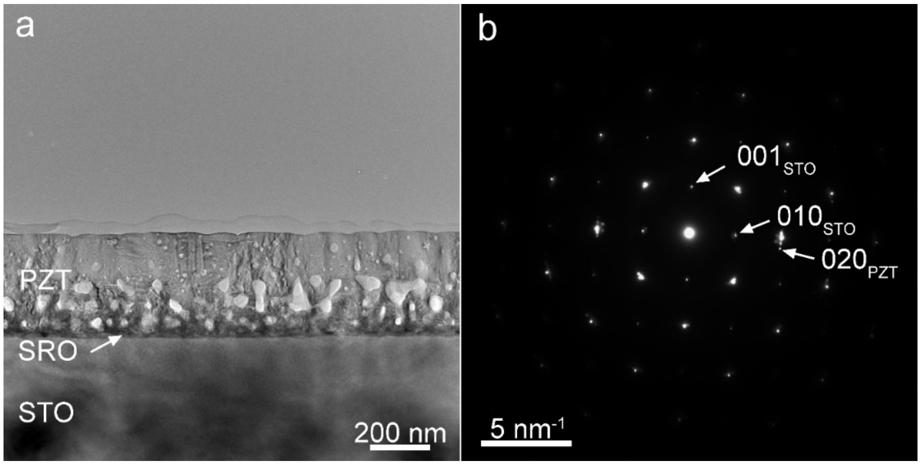

Figure S1. (**a**) TEM image at low magnification of the PZT/SRO/STO structure and (**b**) the corresponding SAED pattern from an area which include both the substrate and the thin films.

2. PFM studies

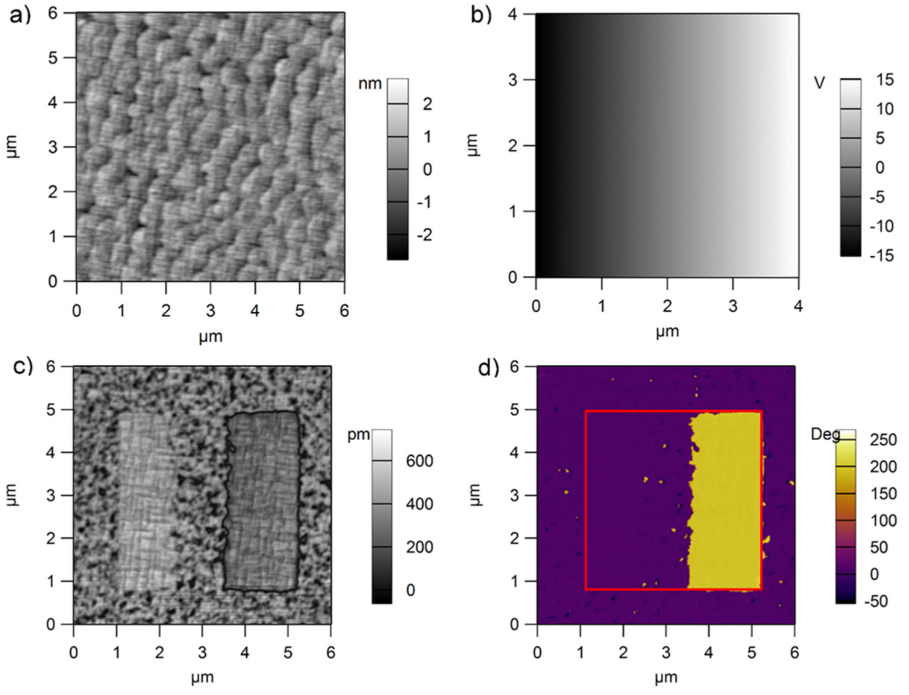

Figure S2. Topography (**a**), poling map (**b**) amplitude (**c**) and phase (**d**) of the PFM signal obtained after poling in the case of the PZT films deposited from the in-house made, pure target on single crystal STO substrate with bottom SRO electrode.

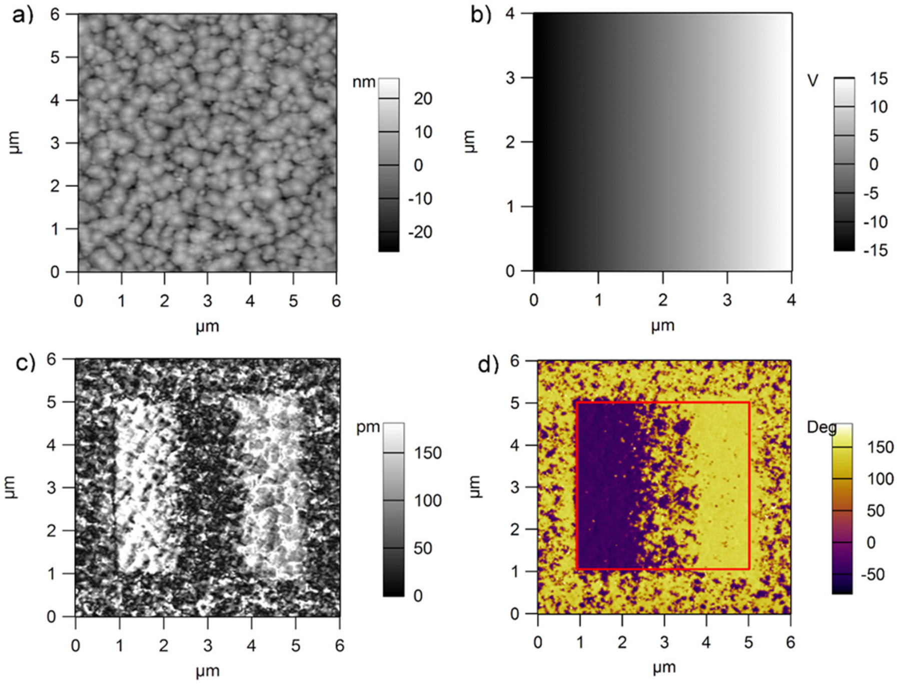

Figure S3. Topography (**a**), poling map (**b**) amplitude (**c**) and phase (**d**) of the PFM signal obtained after poling in the case of the PZT films deposited by sol-gel on single crystal STO substrate with bottom SRO electrode.
